# Thienothiophene-based organic light-emitting diode: synthesis, photophysical properties and application

**DOI:** 10.3762/bjoc.19.137

**Published:** 2023-12-07

**Authors:** Recep Isci, Turan Ozturk

**Affiliations:** 1 Department of Chemistry, Istanbul Technical University, 34469, Maslak, Istanbul, Turkey; 2 TUBITAK UME, Chemistry Group Laboratories, 41470, Gebze, Kocaeli, Turkey

**Keywords:** OLED, organoboron, solution processes, thienothiophene, triphenylamine

## Abstract

A donor–π–acceptor (D–π–A)-type pull–push compound, DMB-TT-TPA (**8**), comprising triphenylamine as donor and dimesitylboron as acceptor linked through a thieno[3,2-*b*]thiophene (TT) π-conjugated linker bearing a 4-MeOPh group, was designed, synthesized, and fabricated as an emitter via a solution process for an organic light-emitting diode (OLED) application. DMB-TT-TPA (**8**) exhibited absorption and emission maxima of 411 and 520 nm, respectively, with a mega Stokes shift of 109 nm and fluorescence quantum yields both in the solid state (41%) and in solution (86%). The optical properties were supported by computational chemistry using density functional theory for optimized geometry and absorption. A solution-processed OLED was fabricated using low turn-on voltage, which had performances with maximum power, current, and external quantum efficiencies of 6.70 lm/W, 10.6 cd/A, and 4.61%, respectively.

## Introduction

In recent years, organic electronics have become very attractive due to their various advantages such as high flexibility, easy designability, low fabrication cost, easy processing and large-scale fabrication [1–4]. Especially in display technology, organic-based materials have found use in many applications such as OLEDs, micro-LEDs, LCDs, lasers, and photodiodes by applying thin film methods and solution processes [5–8]. The performance of organic electronics is based on the active layer composition as well as the fabrication methods and processing parameters. The organic active layers are composed of various aromatic π-conjugated small molecules/polymers including thiophene, anthracene, carbazole, and triphenylamine [9–13].

Thienothiophenes are two annulated thiophene rings having four isomers, among which the most widely used isomer is thieno[3,2-*b*]thiophene (TT) [14–19]. These compounds are electron-rich, flat and electron-delocalized systems, properties that make them promising materials for the construction of conjugated energy-based semiconductors for OLEDs [20–23], perovskite solar cells [24–25], organic field-effect transistors (OFETs) [26–28], capacitors [29–30], hybrid films [31], and photosensitizers [32–34]. Another important π-conjugated unit is triphenylamine (TPA), having an ionization potential of 6.80 eV, which is lower compared to many other organic cores, thus providing a strong electron-donating ability for organic electronic applications [12,35]. Dimesitylboron (DMB), with its unoccupied p-orbital, is an electron-acceptor organoboron compound used in several donor–acceptor systems to provide the system with pull–push interaction [36–37].

In this work, we have designed and synthesized a D–π–A model pull–push fluorophore, DMB-TT-TPA (**8**), having TPA and DMB units as donor and acceptor units that were linked through a 4-MeOPh-substituted TT core as a π-spacer. The photophysical properties of the fluorophore were investigated by spectroscopic methods. Moreover, DMB-TT-TPA (**8**) was fabricated as an emitter for an organic light-emitting diode through a solution process. DMB-TT-TPA (**8**) displayed excellent performance in both device application and photophysical properties, i.e., a maximum solution fluorescence quantum yield of 86% in THF, maximum solid-state fluorescence quantum yield of 41%, maximum current efficiency of 10.6 cd/A, and maximum power efficiency of 6.70 lm/W.

## Results and Discussion

### Design and synthesis

**Scheme 1:**
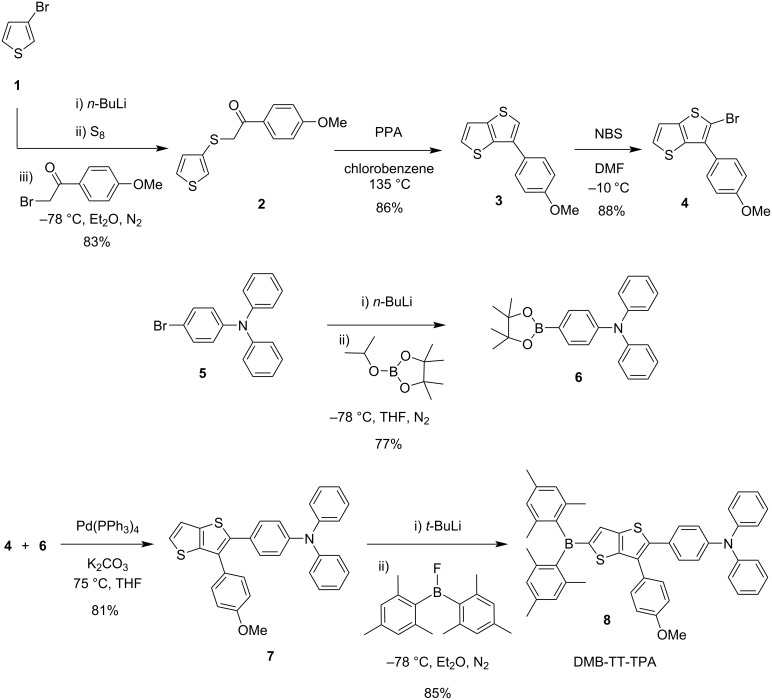
Synthesis of DMB-TT-TPA (**8**).

The OLED fluorophore, DMB-TT-TPA (**8**, Scheme 1), having a donor–π–acceptor (D–π–A) system, was synthesized according to our previously reported methods [20–23,36,38]. The synthesis commenced with the treatment of 3-bromothiophene (**1**) with *n*-butyllithium at −78 °C, followed by the addition of elemental sulfur and subsequent reaction with 2-bromo-1-(4-methoxyphenyl)ethanone to produce compound **2** in 83% yield. The following ring-closure reaction was conducted in the presence of polyphosphoric acid (PPA) in refluxing chlorobenzene to give **3** (TT) in 86% yield. The brominated TT **4** was obtained through selective monobromination of compound **3** using NBS at −10 °C in DMF in 88% yield. The boronated triphenylamine **6** was constructed in a one-pot two-step reaction in 77% yield, by lithiation of 4-bromo-*N*,*N*-diphenylaniline (**5**) with *n*-butyllithium at −78 °C and addition of 2-isopropoxy-4,4,5,5-tetramethyl-1,3,2-dioxaborolane. The Suzuki-coupling reaction of TT **4** with borolane **6** produced the intermediate **7** in 81% yield. The target D–π–A-type fluorophore, DMB-TT-TPA (**8**), was produced by lithiation of **7** and following reaction with dimesitylboron fluoride in 85% yield (Scheme 1).

### Photophysical properties

**Figure 1:**
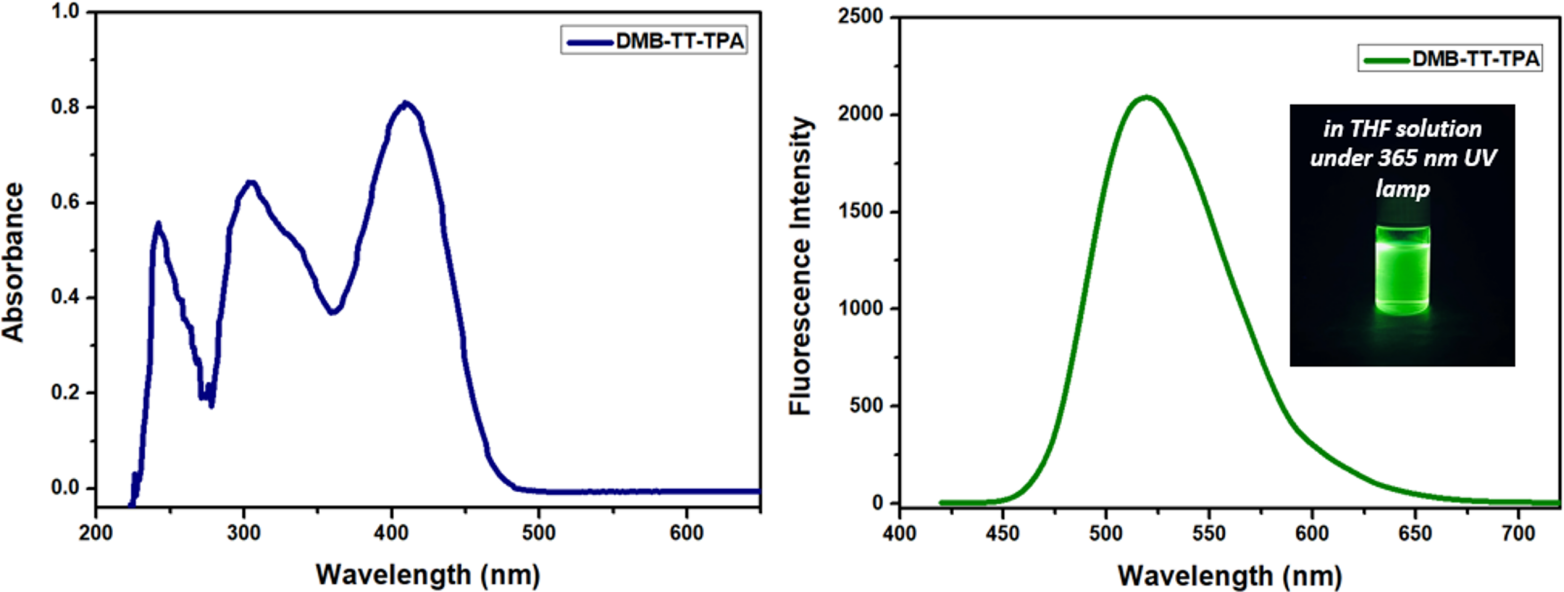
Absorption and emission of DMB-TT-TPA (**8**) in THF. Figure 1 was adapted with permission of Institution of Chemical Engineers (IChemE) and The Royal Society of Chemistry from [38] (“Cationic and radical polymerization using a boron–thienothiophene–triphenylamine based D-π-A type photosensitizer under white LED irradiation”) by A. Suerkan et al., Mol. Syst. Des. Eng., vol. 8, issue 10, © 2023); permission conveyed through Copyright Clearance Center, Inc. This content is not subject to CC BY 4.0.

The UV–vis absorption and fluorescence spectra of DMB-TT-TPA (**8**) were recorded in THF (Figure 1 and Table 1) [38]. It showed maximum absorption and emission wavelengths of 411 and 520 nm (excitation at λ_max_), respectively, leading to a mega Stokes shift (>100 nm) of 109 nm, which could be explained to be due to a fast relaxation from the excited state to the ground state as a result of a powerful intramolecular energy transfer between the TPA and boron groups through the thieno[3,2-*b*]thiophene (TT) core. The optical band gap (*E*_optic_) of DMB-TT-TPA (**8**) was calculated to be 2.52 eV from the onset wavelength of the absorption spectrum at 491 nm. The compound demonstrates high quantum efficiencies in the solid-state and in solution (THF) of 41 and 86%, respectively. The considerable quantum efficiencies pointed out that DMB-TT-TPA (**8**) is among the best D–π–A modal fluorophores suitable for an OLED application. Moreover, the photophysical properties of DMB-TT-TPA (**8**) were investigated through time-resolved fluorescence studies (390 nm laser source in THF). The fluorescence lifetime (τ) of DMB-TT-TPA (**8**) exhibited a mono-exponential profile having a 3.20 ns fluorescence decay pattern (Figure S1 in Supporting Information File 1), demonstrating a strong pull–push interaction in steady-state time resolved fluorescence performance.

**Table 1:** Photophysical data of DMB-TT-TPA (**8**) [38].

Compound	UV_max_^a^ (nm)	UV_onset_ (nm)	Fl_max_^a^ (nm)	Δν^b^ (cm^−1^)	*E*_optic_^c^ (eV)	Φ_solid_^d (^%)	Φ_sol_^e^ (%)

DMB-TT-TPA	411	491	520	5100	2.52	41	86

^a^Absorption and fluorescence maxima in THF. ^b^Stokes shift (cm^−1^) Δν = 1/λ_max_ − 1/λ_em_. ^c^*E*_optic_ from the onset of the absorption spectrum. ^d^Solid-state quantum yield. ^e^Solution-state quantum yield in THF.

### OLED application

**Figure 2:**
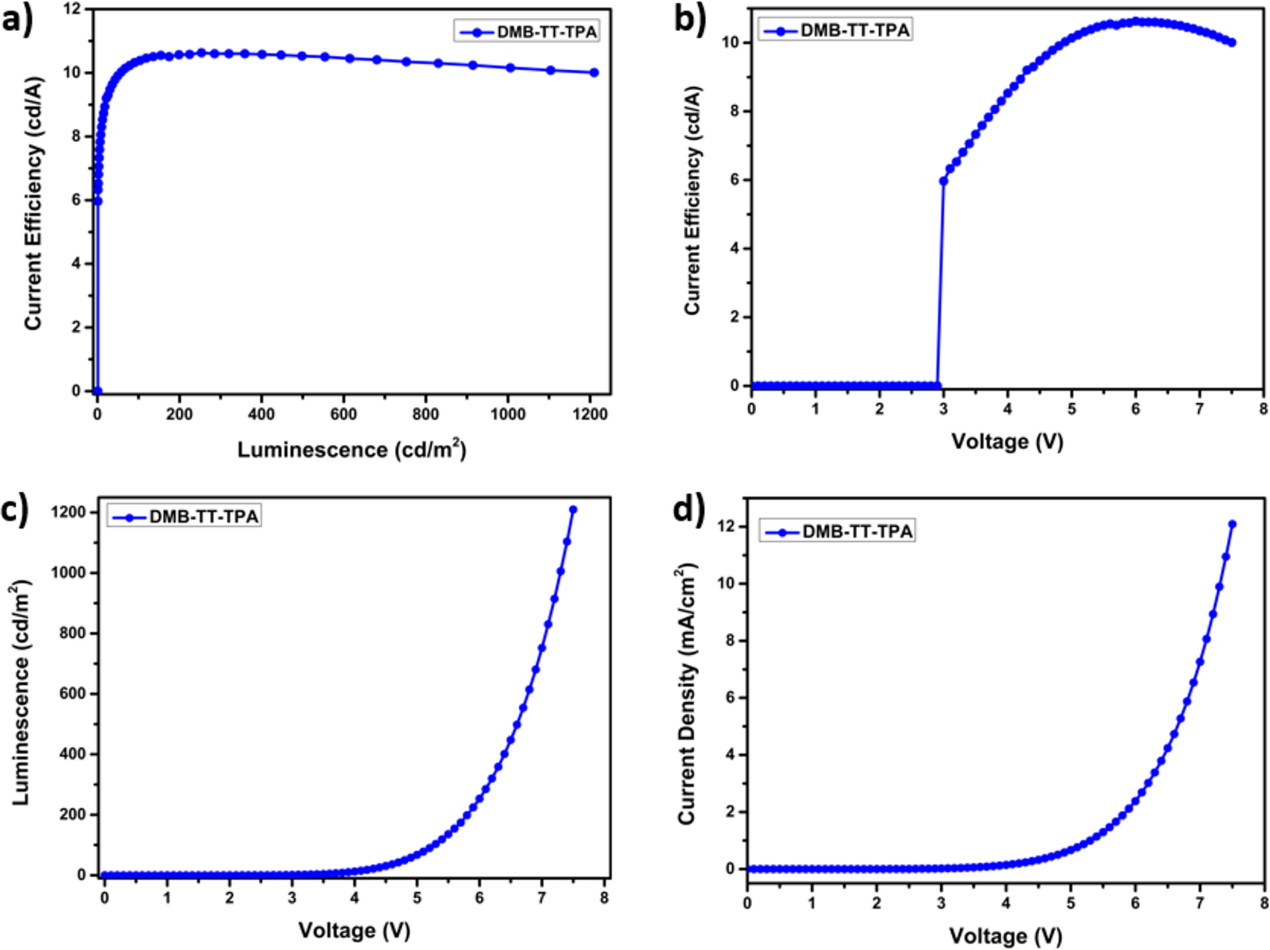
(a) Current efficiency–luminance, (b) current efficiency–voltage, (c) luminance–voltage, and (d) current density–voltage characteristics of DMB-TT-TPA (**8**).

**Figure 3:**
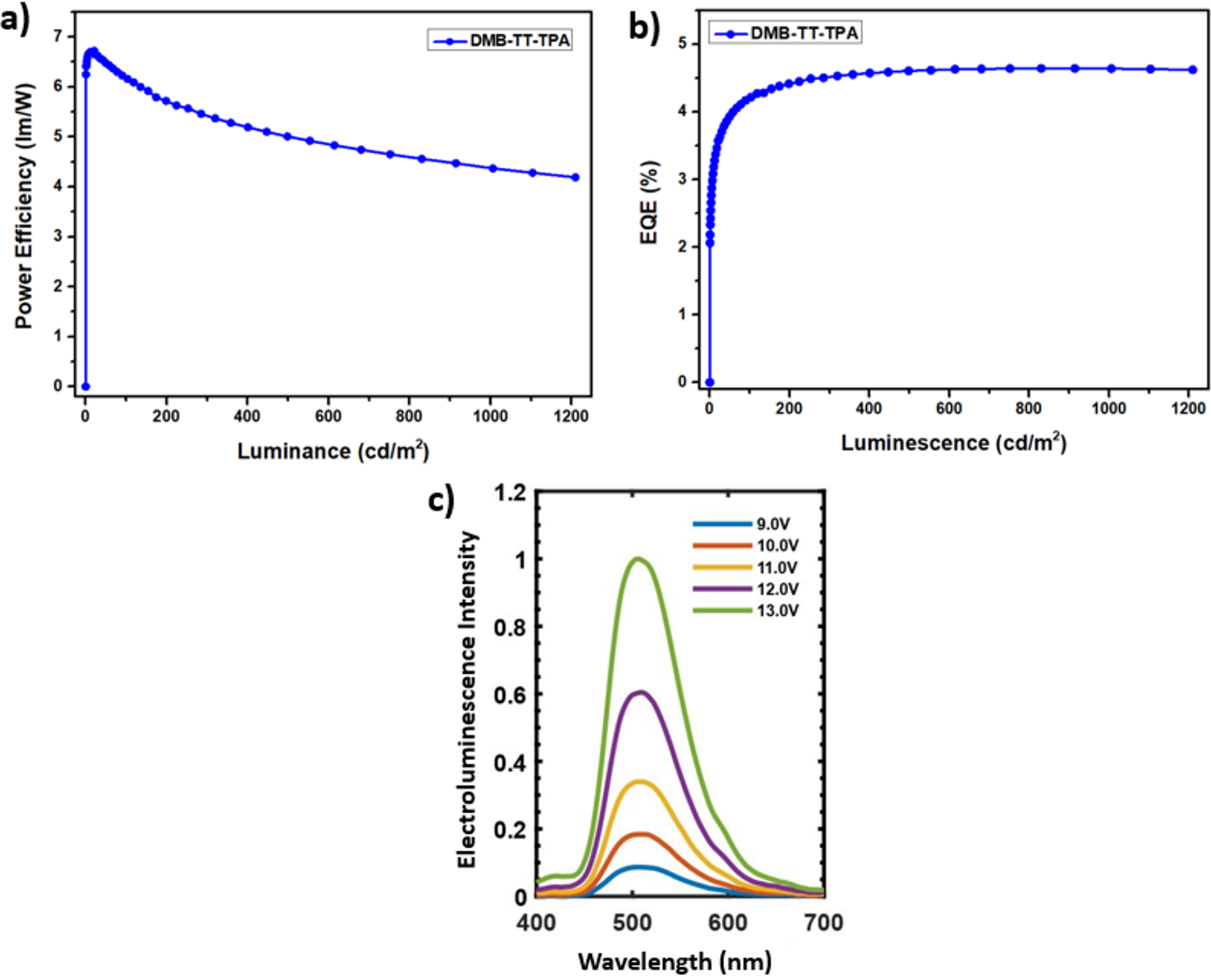
(a) Power efficiency–luminance, (b) external quantum efficiency–luminescence, (c) electroluminescence–wavelength characteristics of DMB-TT-TPA (**8**).

An OLED was fabricated using a standard conventional device architecture of ITO/PEDOT:PSS/TFB/TAPC:TCTA:emitter (DMB-TT-TPA (**8**))/TPBi/LiF/Ca/Ag, where TFB, TCTA/TAPC, and TPBi acted as hole transport, hole transporting host, and electron transport materials, respectively (Figure S2 in Supporting Information File 1). The current efficiency–luminance–voltage (J–L–V) graph and power efficiency (PE), external quantum efficiency (EQE), and electroluminescence curves are depicted in Figure 2 and Figure 3, respectively. Although DMB-TT-TPA (**8**) was synthesized and OLED performance was examined in our previous study [23], a different device architecture and method, i.e., solution processing, was used in this study. In the previous study, the OLED of DMB-TT-TPA (**8**) was explained to demonstrate performance with the turn-on voltage, external quantum efficiency (EQE), and highest luminescence efficiency of 4.6 V, 0.15% and 0.40 cd/A, respectively, using a thermal evaporation method. On the other hand, in this study, the OLED of DMB-TT-TPA (**8**), prepared using a solution processing method, showed a low turn-on voltage (*V*_on_) of 2.90 V, a max current efficiency (CE_max_) of 10.6 cd/A, a max luminance of 752 cd/m^2^, a max power efficiency (PE_max_) of 6.70 lm/W, and an external quantum efficiency (EQE) of 4.61%, along with a green emitting luminescence at 512 nm (Table 2). According to the CIE color space chromaticity diagram, the device was located at the coordinates of 0.16 and 0.51. The obtained EL results are in good agreement with the fluorescence characteristic of DMB-TT-TPA (**8**). Additionally, OLED performances were significantly increased compared to the previous study [23]. In terms of the TT chemistry, the device results reached remarkable values for donor–π–acceptor-type solution processable emitters within the donor–acceptor family [39–42]. This approach also supports that the solution-processable OLED application is a perfectly suitable device preparation for DMB-TT-TPA (**8**).

**Table 2:** Photophysical data of DMB-TT-TPA (**8**).

compound	*V*_on_^a^ (V)	CE^b^ (cd/A)	L^c^ (cd/m^2^)	λ_EL_^d^ (nm)	EQE^e^ (%)	PE_max_^f^ (lm/W)	CIE^g^ (x, y)

DMB-TT-TPA	2.9	10.6	752	512	4.61	6.70	(0.16, 0.51)

^a^Turn-on voltage, recorded at the luminance of 1 cd·m^2^. ^b^Maximum current efficiency. ^c^Maximum luminance. ^e^Maximum electroluminescence wavelength. ^d^Maximum current efficiency. ^e^Maximum external quantum efficiency. ^f^Maximum power efficiency. ^g^Chromaticity coordinates according to the CIE 1931 diagram.

### Thermal properties

**Figure 4:**
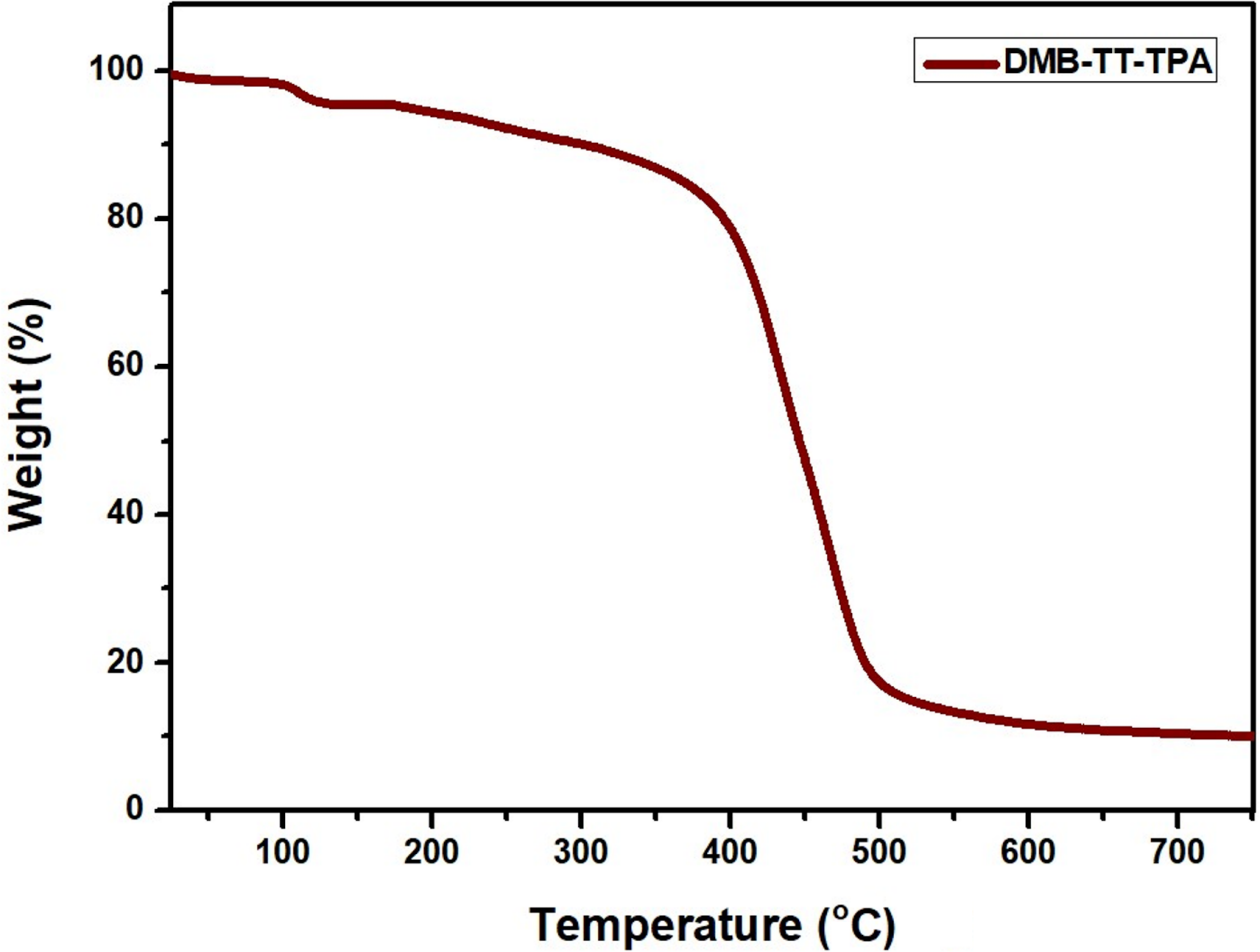
Thermal gravimetric analyses (TGA) of DMB-TT-TPA (**8**).

The thermal properties of DMB-TT-TPA (**8**) were investigated through thermal gravimetric analysis (TGA) at 750 °C at a heating rate of 10 °C min^−1^ under N_2_ atmosphere (Figure 4). The initial mass loss (5%) around 120 °C could be due to residual water and/or solvent. The highest decomposition was observed at around 405 ^o^C and 14% of DMB-TT-TPA (**8**) remained without ash up to 750 °C, indicating that the compound has an excellent thermal stability. The high thermal stability is profitable for the preparation of stable and durable OLED devices.

### Computational chemistry

**Figure 5:**
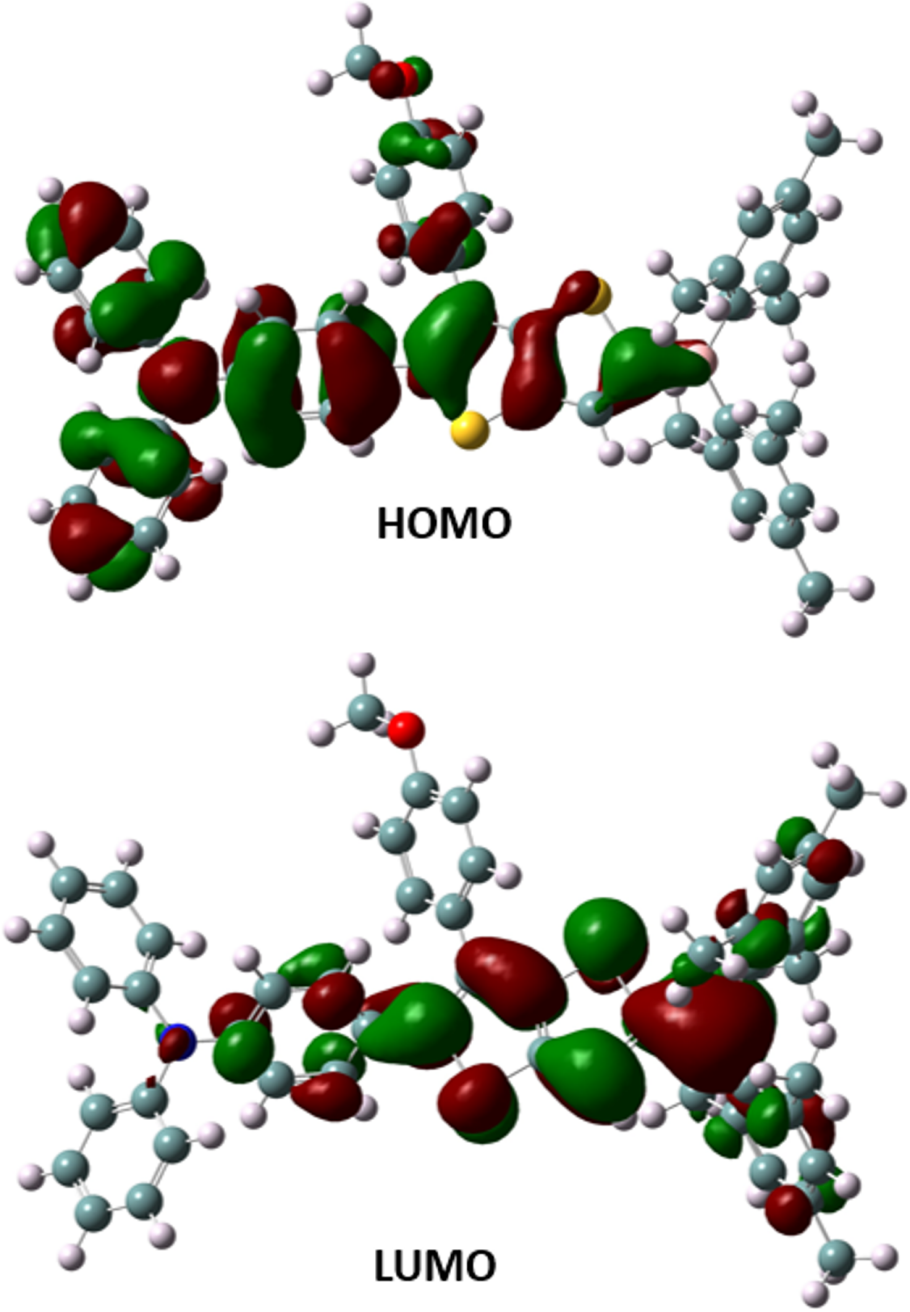
HOMO and LUMO diagrams calculated at the B3LYP/6-31G (d,p) level of theory.

Ground-state geometry optimization of DMB-TT-TPA (**8**) was performed using density functional theory (DFT) calculations with the Gaussian 16 software at the B3LYP/6-31G (d,p) level (Figure S3 in Supporting Information File 1) [23,43]. The highest occupied molecular orbital (HOMO) and the lowest unoccupied molecular orbital (LUMO) energy levels were calculated to be −4.93 and −1.83 eV, respectively (Figure 5). While the HOMO electrons were distributed mainly on the triphenylamine and TT units, the LUMO was found to be delocalized through the dimesitylboron and TT ring, the results being in line with the experimental values of our previous study [23].

On the basis of the optimized ground-state geometry, time-dependent DFT (TD-DFT) calculations were conducted in THF to investigate the absorption properties and theoretical band gap (Table 3). The optical band gap value (*E*_optic_) was calculated to be 2.06 eV, considering the λ_onset_ (605 nm) of the UV–vis curve. The calculated absorption maximum was centered at 470 nm (Figure S4 in Supporting Information File 1), which was found to be in a good agreement with the experimentally determined UV–vis spectrum.

**Table 3:** The HOMO and LUMO energy levels and absorption values calculated by TD-B3LYP/6-31G (d,p) level of theory.

Compound	HOMO (eV)	LUMO (eV)	λ_max_ (nm)	λ_onset_ (nm)	*E*_optic_ (eV)

DMB-TT-TPA (**8**)	−4.93	−1.83	470	605	2.06

## Conclusion

A small fluorophore molecule, DMB-TT-TPA (**8**), containing dimesitylboron as an acceptor and triphenylamine as a donor linked through a thieno[3,2-*b*]thiophene core having a 4-MeOPh group, was designed as a D–π–A model and synthesized in 85% yield. Its photophysical properties were investigated by UV–vis and fluorescence spectroscopy. The obtained experimental results were found to be in good agreement with computational investigations. An OLED fabrication, where DMB-TT-TPA (**8**) was employed as an emitter, showed a maximum luminescence efficiency of 752 cd/m^2^, a maximum external quantum efficiency of 4.61%, a maximum power efficiency of 6.70 lm/W, and a maximum current efficiency of 10.6 cd/A on 2.9 V turn on voltage with CIE coordinates of 0.16 and 0.51 at λ_EL_ = 512 nm. The OLED, optical and thermal properties indicated that the composition of thienothiophene, triphenylamine, and boron is a highly suitable combination for fluorescent organic electronics in display technology.

## Experimental

### General methods

^1^H and ^13^C NMR spectra were recorded on a Varian model NMR spectrometer (500 and 126 MHz) and chemical shift values are reported in ppm downfield from tetramethylsilane (TMS). UV–vis absorption spectra were obtained using a HITACHI U-0080D spectrophotometer. Fluorescence spectra were recorded on a HITACHI F-4500 fluorescence spectrophotometer. Time-resolved fluorescence studies were performed on a Horiba, FL3-2IHR fluorescence spectrophotometer. Solid-state and solution-state quantum yields were measured using a Hamamatsu Quantaurus-QY Absolute PL Quantum Yield Spectrometer. Thermal gravimetric analysis (TGA) was performed on a PerkinElmer Diamond TA/TGA with a heating rate of 10 °C min^−1^ under nitrogen flow.

### Materials

3-Bromothiophene (97%, Across), 2-bromo-4′-methoxyacetophenone (97% Merck), *N*-bromosuccinimide (Sigma-Aldrich), polyphosphoric acid (PPA, 115% H_3_PO_4_ basis, Sigma-Aldrich), *n*-butyllithium (2.5 M in hexanes, Sigma-Aldrich), sodium sulfate (Merck), 4-bromotriphenylamine (Sigma-Aldrich), dimesitylboronfluoride (90%, Sigma-Aldrich), 4,4,5,5-tetramethyl-1,3,2-dioxaborolane (Sigma-Aldrich), tetrakis(triphenylphosphine)palladium(0) (Pd(PPh_3_)_4_, 99%, Sigma-Aldrich), were used as received. Diethyl ether and THF were dried over metallic sodium. Dimethylformamide (HPLC grade) was stored over activated molecular sieves (4 Å). Dichloromethane (Aldrich), methanol (Merck), and sodium carbonate (Merck) were used as received. Compounds **2**–**6** were synthesized following our previous reports [20–23,44–47]. The characterization data of **7** and **8** are compatible with the published data in ref. [23].

### Synthesis of **7**

Synthesized as described in [23]. To a mixture of thienothiophene **4** (250 mg, 0.770 mmol) and borolane **6** (320 mg, 0.845 mmol) dissolved in THF (25 mL) and degassed for 45 min with N_2_ was added K_2_CO_3_ (2.5 mL, 2.5 M) and Pd(PPh_3_)_4_ (0.077 mmol). The mixture was then saturated with N_2_, the reaction flask sealed and the mixture stirred at 75 °C for 48 h. Afterwards, the reaction mixture was filtered through celite eluting with CH_2_Cl_2_, extracted with CH_2_Cl_2_/water, and the organic phase was washed with sodium carbonate solution (10%) and water, dried over sodium sulfate, filtered, and the solvent was evaporated under reduced pressure. The crude product was purified by column chromatography eluting with *n*-hexane/CH_2_Cl_2_ 4:1 to obtain the title compound **7** (300 mg, 81%) as a white powder. Mp 141–142 °C; ^1^H NMR (500 MHz, CDCl_3_) δ 7.42 (d, *J* = 8.8 Hz, 2H), 7.35 (d, *J* = 5.2 Hz, 1H), 7.28 (t, *J* = 8.7 Hz, 5H), 7.20 (d, *J* = 8.7 Hz, 2H), 7.13 (d, *J* = 7.6 Hz, 4H), 7.05 (t, *J* = 7.3 Hz, 2H), 6.95 (d, *J* = 8.7 Hz, 2H), 6.92 (d, *J* = 8.8 Hz, 2H), 3.86 (s, 3H); ^13^C NMR (126 MHz, CDCl_3_) δ 158.89, 147.36, 147.17, 142.04, 139.51, 135.73, 130.12, 129.87, 129.29, 128.34, 127.96, 125.86, 124.80, 123.24, 122.53, 119.81, 114.14, 55.22.

### Synthesis of DMB-TT-TPA (**8**)

Synthesized as described in [23]. To a solution of compound **7** (200 mg, 0.410 mmol) in dry THF (50 mL) was added *tert*-butyllithium (0.3 mL, 1.7 M, 0.490 mmol) dropwise at −78 °C under a nitrogen atmosphere over a period of 45 min. Then, dimesitylborofluoride (130 mg, 0.490 mmol) was added rapidly. The mixture was further stirred at −78 °C for 1 h, then, allowed to warm to room temperature and stirring was continued overnight. The solution was extracted with dichloromethane, and the organic layer was washed with Na_2_CO_3_ solution (10%) and water. The organic layer was dried over Na_2_SO_4_, filtered and the solvent was evaporated under reduced pressure. The crude product was purified by flash column chromatography eluting with a mixture of *n*-hexane/CH_2_Cl_2_ 3:1 and then crystallized from ethanol to give the title compound DMB-TT-TPA (**8**) as a yellow powder in 85% yield (256 mg). Mp 165–166 °C; ^1^H NMR (500 MHz, CDCl_3_) δ 7.59 (s, 1H), 7.39 (d, *J* = 8.8 Hz, 2H), 7.28 (d, *J* = 7.8 Hz, 4H), 7.19 (d, *J* = 8.7 Hz, 2H), 7.12 (d, *J* = 7.6 Hz, 4H), 7.05 (t, *J* = 7.3 Hz, 2H), 6.93 (d, *J* = 8.7 Hz, 2H), 6.87 (d, *J* = 8.8 Hz, 2H), 6.84 (s, 4H), 3.82 (s, 3H), 2.32 (s, 6H), 2.17 (s, 12H); ^13^C NMR (126 MHz, CDCl_3_) δ 158.92, 153.46, 151.26, 147.60, 147.20, 143.95, 141.05, 140.90, 138.50, 137.96, 132.59, 130.25, 129.86, 129.49, 129.33, 128.14, 127.85, 127.57, 125.01, 123.45, 122.08, 114.12, 55.23, 23.54, 21.22.

## Supporting Information

File 1 General experimental device methods, life time spectra, theoretical computation data, ^1^H and ^13^C NMR spectra.
