# Supplementary material for: Thienothiophene-based organic light-emitting diode: synthesis, photophysical properties and application
Source: Beilstein J Org Chem. 2023 Dec 7;19:1849–57. doi: 10.3762/bjoc.19.137 (PMC10714501; doi:10.3762/bjoc.19.137)
Supplement: File 1 — General experimental device methods, life time spectra, theoretical computation data, 1H and 13C NMR spectra. [file Beilstein_J_Org_Chem-19-1849-s001.pdf]

BEILSTEIN JOURNAL OF ORGANIC CHEMISTRY

Supporting Information

for

Thienothiophene-based organic light-emitting diode: synthesis, photophysical properties and application

Recep Isci and Turan Ozturk

Beilstein J. Org. Chem. **2023,** *19,* 1849–1857. doi:10.3762/bjoc.19.137

General experimental device methods, life time spectra, theoretical computation data, ^{1}H and ^{13}C NMR spectra

License and Terms: This is a supporting information file under the terms of the Creative Commons Attribution License (https://creativecommons.org/licenses/by/4.0). Please note that the reuse, redistribution and reproduction in particular requires that the author(s) and source are credited and that individual graphics may be subject to special legal provisions.
The license is subject to the *Beilstein Journal of Organic Chemistry* terms and conditions: (https://www.beilstein-journals.org/bjoc/terms)

Table of contents

1. Fluorescence lifetime……………………………………………………………...S1

2. General experimental device methods………………………………………..S1

3. Computational data…………………………………………………...…...S2

4. NMR spectra…………………………………………………………………......S4

1. Fluorescence lifetime

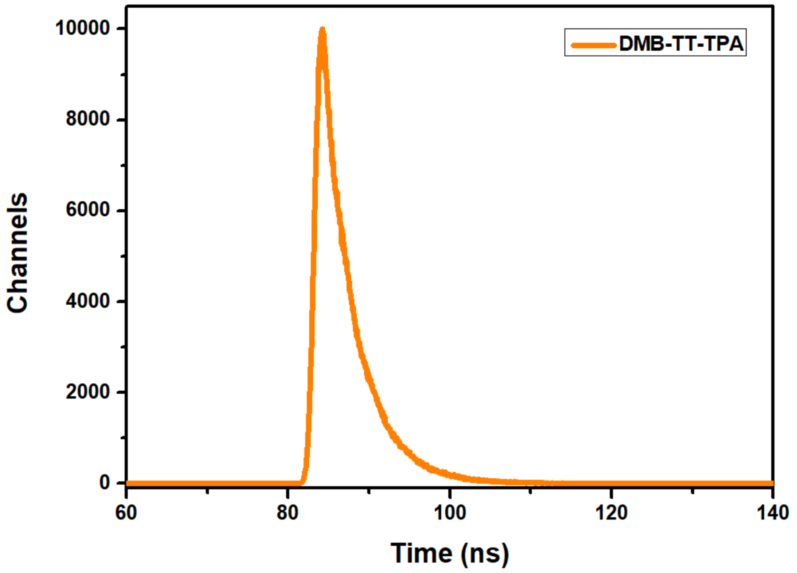

Figure S1: Fluorescence lifetime decay pattern of the DMB-TT-TPA (**8**).

2. General experimental device methods

TFB (Cambridge Display Technology Ltd.) solution was prepared by dissolving TFB in toluene in 2 mg/mL concentration. The active layer solution was prepared by controlling the host material concentration fixed at 15 mg/mL and blending the emitter at 10 wt % in chlorobenzene. Prepatterned ITO substrates were rinsed in an ultrasonic bath with acetone, isopropyl alcohol, Hellmanex III, and deionized water before deposition of top layers. PEDOT:PSS and TFB were deposited on ITO sequentially via spin-coating at 2500 and 1000 rpms, respectively. Then, the prepared active layer was spin-coated on annealed TFB (180 °C for 1 h in a nitrogen glovebox) at 2000 rpms to form a 50 nm film. Afterwards, TPBi, LiF, and Ca/Ag were thermally evaporated onto the organic layer under vacuum level of ≈ 5×10^{-7} mbar. J–V–L characterization (pixel area = 0.045 cm^2) was performed using a Keithley 2400 and Konica Minolta LS-110 Luminance Meter. OLED emission profile was assumed to be Lambertian. The EL

spectrum was measured using an Ocean Optics USB 2000 charge-coupled device spectrophotometer.

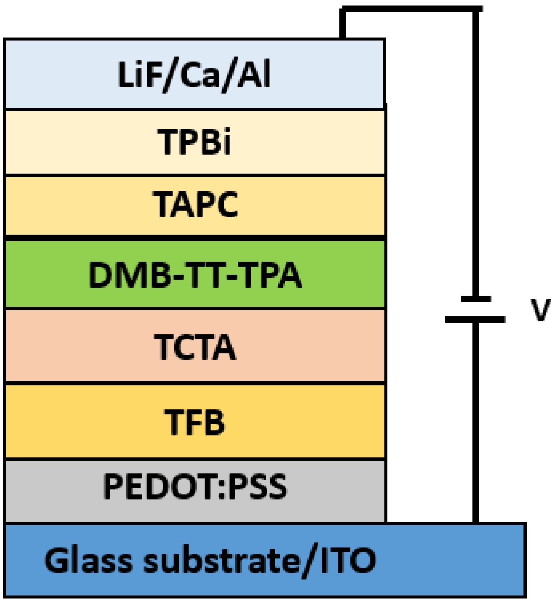

Figure S2: Schematic illustration of the device configuration.

3. Computational data

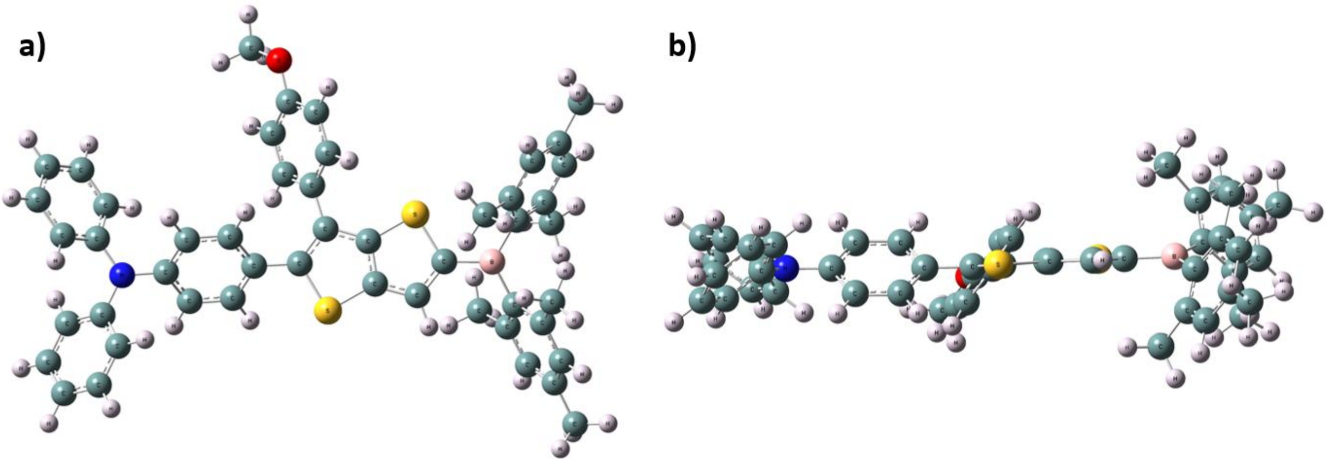

Figure S3: Optimized geometry of DMB-TT-TPA (**8**) of a) top view and b) side view.

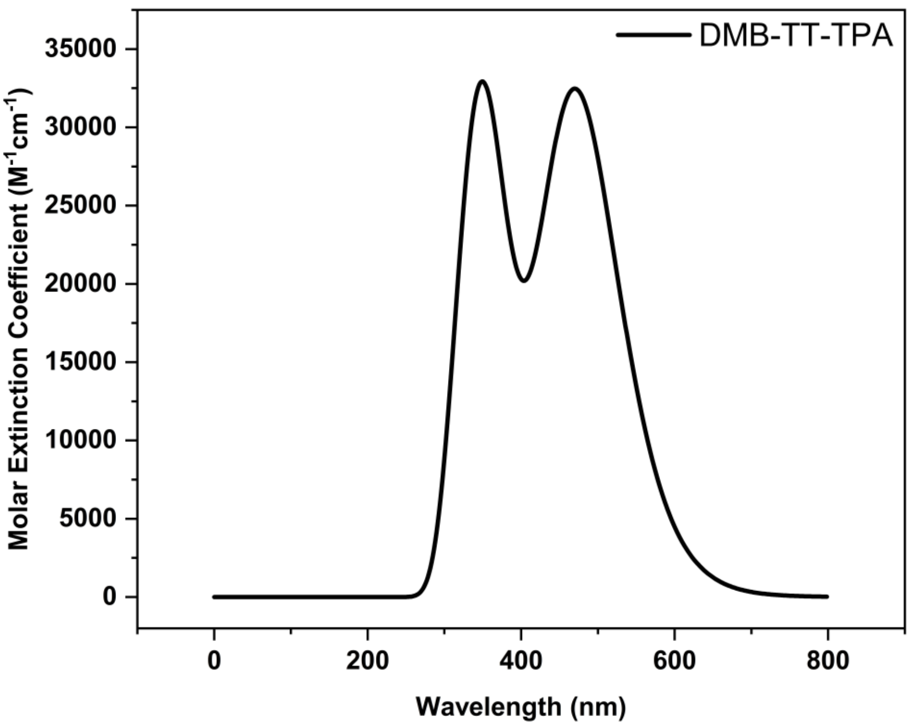

Figure S4: Theoretical UV–vis spectrum of DMB-TT-TPA (**8**).

4. NMR spectra

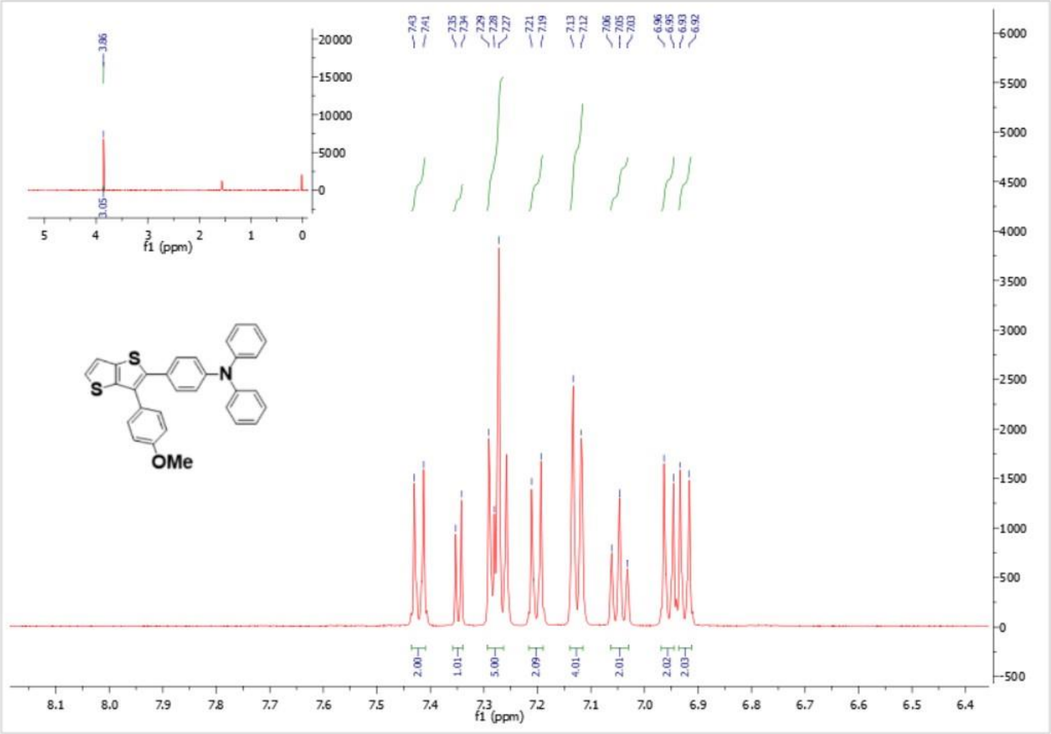

Figure S5: ^{1}H NMR spectrum of **7**.

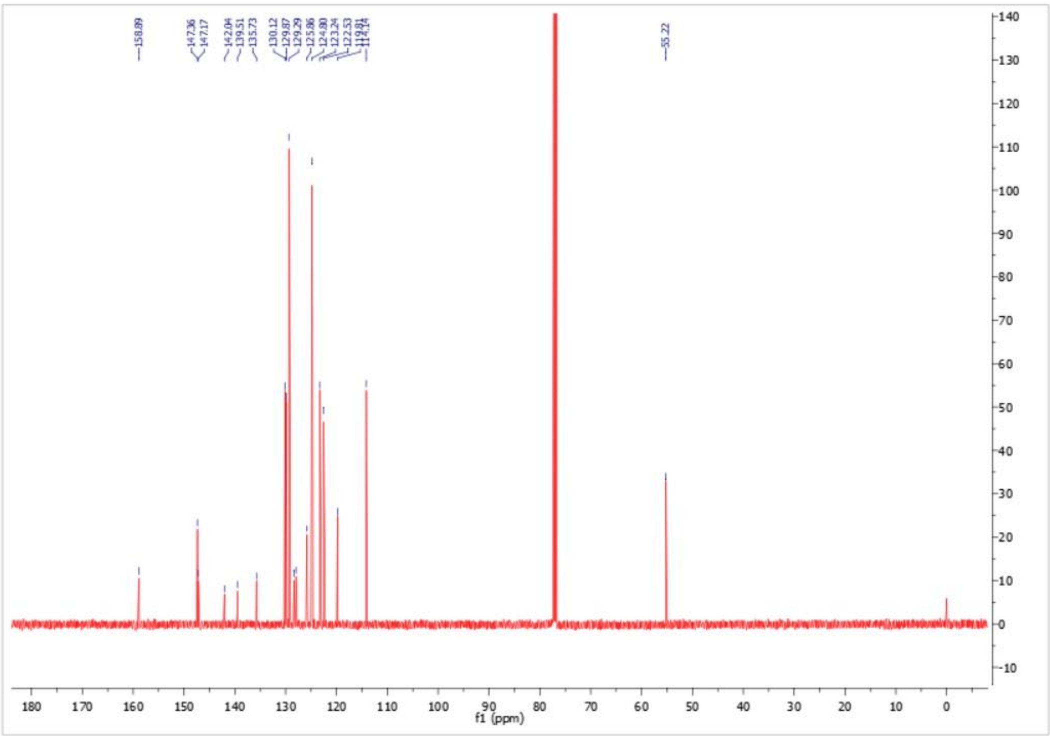

Figure S6: ^{13}C NMR spectrum of **7**.

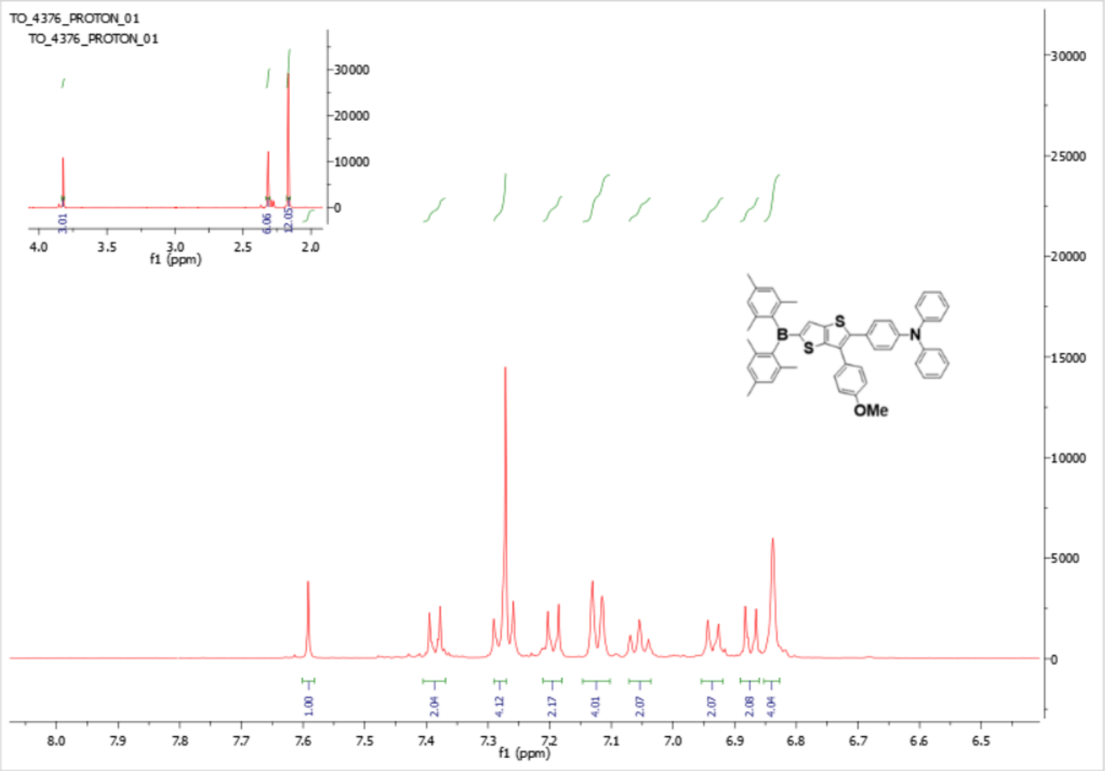

Figure S7: ^{1}H-NMR spectrum of DMB-TT-TPA (**8**).

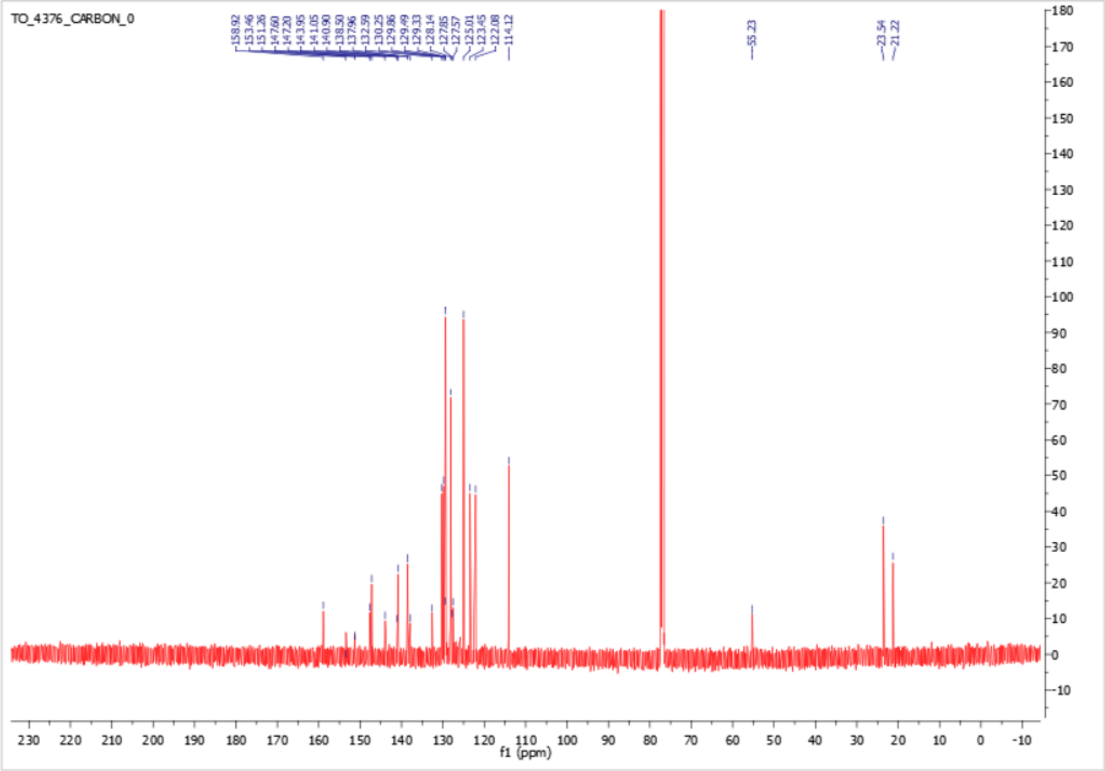

Figure S8: ^{13}C NMR spectrum of DMB-TT-TPA (**8**).
